# Development of Site-Specific PEGylated Granulocyte Colony Stimulating Factor With Prolonged Biological Activity

**DOI:** 10.3389/fbioe.2020.572077

**Published:** 2020-11-19

**Authors:** Monika Kumari, Girish Sahni, Sonal Datta

**Affiliations:** Council of Scientific and Industrial Research, Institute of Microbial Technology, Chandigarh, India

**Keywords:** G-CSF, PEGylated G-CSF, site-specific PEGylation, prolonged biological activity of G-CSF, neutropenia, cancer chemotherapy

## Abstract

Currently, amino-terminal PEGylated human granulocyte colony stimulating factor (huG-CSF) is used to prevent and treat neutropenia. Although huG-CSF has been used as a drug for more than 20 years, it has three significant drawbacks: (i) it relies on PEG aldehyde for PEGylation of the alpha-amino group of the first amino acid, and this leads to non-specific PEGylation of the epsilon amino group of lysine residues within the G-CSF; (ii) longer-acting G-CSF variants are desirable to reduce the risk of chemotherapy-associated neutropenia; and (iii) G-CSF cannot be administered on the day of chemotherapy. In an attempt to overcome the above drawbacks, we engineered cysteine variants of G-CSF to facilitate the maleimide PEG-based site-specific PEGylation that leads to a highly homogenous PEGylated product. Importantly, we have demonstrated that 20 kDa thiol-reactive PEG conjugated by maleimide chemistry to the Cys2 G-CSF variant exhibits leukocyte proliferative activity similar to that of the commercially available G-CSF conjugated with aldehyde PEG in a neutropenia mice model. Moreover, we have demonstrated that PEGylation of the cysteine variant of huG-CSF with higher molecular weight PEGs, such as 30 kDa PEG and 40 kDa PEG, leads to significantly prolonged leukocyte proliferation activity compared to the variant conjugated with 20 kDa PEG. Importantly, even a half-dose of the engineered variant conjugated with 40 kDa PEG exhibited significantly longer biological activity than the commercially available 20 kDa PEGylated huG-CSF. Finally, we have demonstrated that administration of the engineered variant conjugated with 40 kDa PEG on the day of administration of cyclophosphamide for inducing neutropenia in mice can alleviate neutropenia through leukocyte proliferation. In summary, this study provides the design of site-specific PEGylated huG-CSF variants with improved therapeutic potential. It opens the possibility of long-acting and same-day prophylactic administration of G-CSF after chemotherapy drug regimens. These results may pave the way for the development of potential G-CSF derivatives possessing longer half-lives and favorable clinical attributes.

## Introduction

Human granulocyte colony stimulating factor (huG-CSF) is a 19 kDa cytokine that stimulates the proliferation, maturation, and functional activation of the cells in the granulocyte lineage (Anderlini et al., 1996; Welte et al., 1996). HuG-CSF was approved by the Food and Drug Administration (FDA) of the United States to prevent and treat neutropenia, for bone marrow transplantation, and to mobilize peripheral blood progenitor cells for transplantation as well as for blood banking (Nemunaitis, 1997; Nervi et al., 2006). In fact, G-CSF’s prophylactic administration is recognized as a critical factor behind the success of chemotherapy in cancer treatment (Crawford et al., 2004; Lyman, 2006). G-CSF is cleared from the human body through various means, including receptor-mediated endocytosis followed by its degradation (Kuwabara et al., 1996), renal clearance (Kuwabara et al., 1995), and enzymatic degradation mechanism (El Ouriaghli et al., 2003). Therefore, G-CSF has a short circulation half-life of about 3.5 h (Ng and Tang, 2013). This necessitates the administration of daily injections to maintain the effective concentration in the body to combat neutropenia.

**FIGURE 1:**
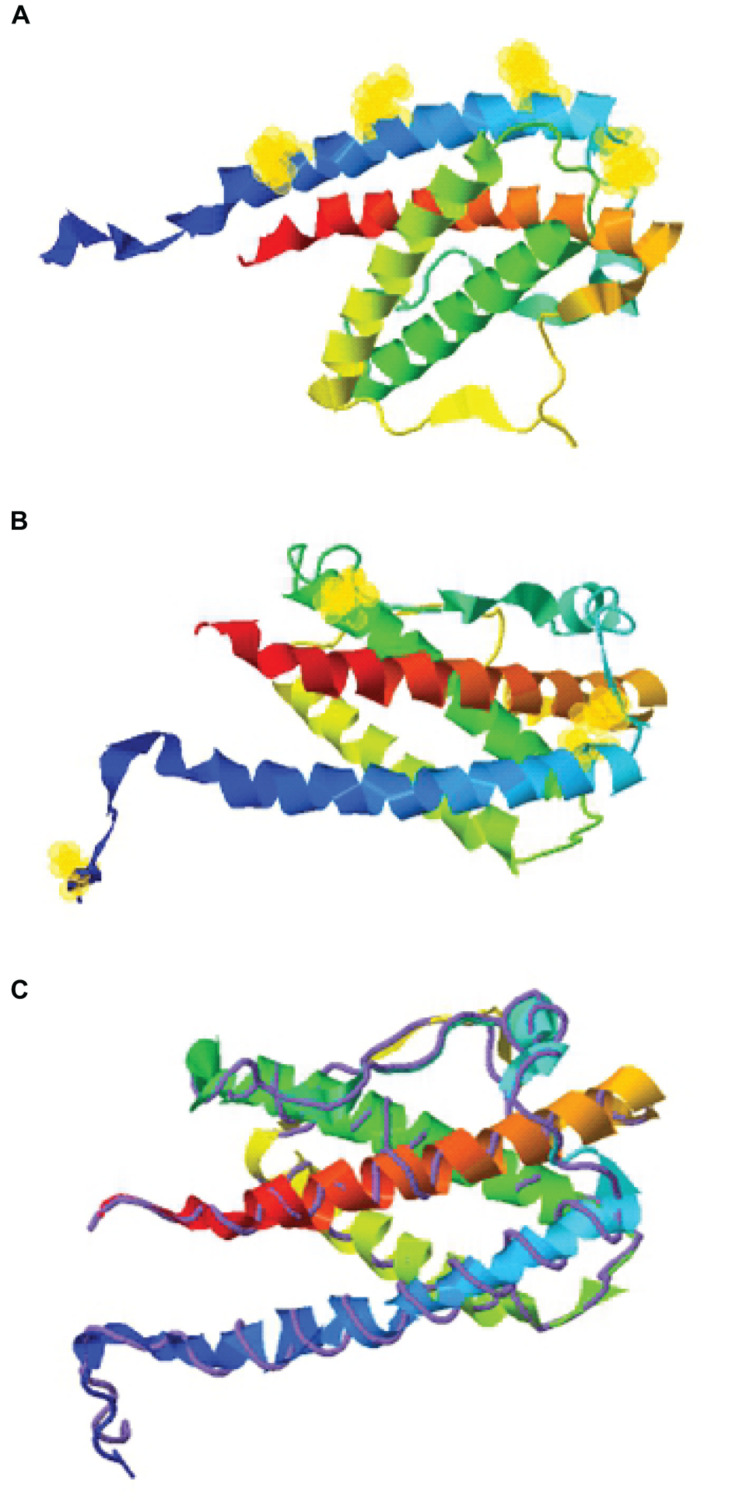
Structure assembly simulation of G-CSF using I-TASSER webserver. **(A)** The position of four lysine residues (at positions 16, 23, 34, and 40) in G-CSF structure by selection halos. **(B)** Cysteine position in the Cys2 variant and four indigenous cysteines involved in two disulfide bonds in the Cys2 G-CSF variant by selection halos. **(C)** The structure assembly simulation using the I-TASSER TM-align structural alignment program to compare the structures of the Cys2 variant (shown in multicolor ribbon cartoon) and human G-CSF (displayed in purple backbone trace). Helix A, B, C, and D are shown in blue, green, yellow, and red, respectively. Lysine and cysteine positions are depicted with yellow halos.

Several approaches have been employed to increase the serum half-life of recombinant huG-CSF. These include conjugation with polyethylene glycol (PEG), known as PEGylation (Molineux, 2004), conjugation with the sialic acid (DeFrees et al., 2006), attachment with the human serum albumin (Paige et al., 1995; Halpern et al., 2002), dimerization of G-CSF (Fidler et al., 2011), a fusion of G-CSF with Fc Domain (Cox et al., 2014), circularization of G-CSF (Miyafusa et al., 2017), conjugation with transferrin (Chen et al., 2010), etc., However, PEGylation has emerged as the method of choice to increase serum half-life and reduce G-CSF’s immunogenicity (Molineux, 2003). PEGylated G-CSF has significantly improved serum half-life (up to 42 h) and is thus administered once-per-cycle of chemotherapy compared to the daily dose of G-CSF (Crawford, 2003). Given these benefits, in 2002, the FDA approved the administration of PEGylated huG-CSF as a prophylactic and therapeutic drug (Molineux, 2004). PEGylation increases the serum half-life of therapeutic proteins primarily through increasing hydrodynamic radii and thereby reducing renal clearance. Besides, PEGylation also masks the protein’s surface to protect it from proteases, antibodies, and antigen processing cells, thus increasing half-life. Furthermore, PEG imparts favorable attributes on the polypeptides to improve their biological distribution and solubility (Pasut and Veronese, 2009). To increase the *in vivo* half-life of G-CSF, 20 kDa methoxy PEG propionaldehyde was conjugated to the alpha-amino group of the first amino acid residue of huG-CSF to make PEGylated G-CSF (Arvedson et al., 2015). Although this method results in efficient PEGylation of G-CSF and is used to PEGylate the currently available commercial G-CSF drug, there are inherent disadvantages associated with this method. This method’s usage leads to a heterogeneous mixture of mono-PEGylated G-CSF due to PEGylation at the epsilon amino group of lysine residues present in G-CSF in addition to PEGylation at the N-terminal amino group. G-CSF contains four lysine residues that could also be PEGylated in an aldehyde-based PEGylation strategy (Figure 1A). Notably, some of the lysine residues are located in regions implicated in binding with the G-CSF receptor (Yamasaki et al., 1994; Tamada et al., 2006). Such heterogeneous populations of drug molecules create difficulty in physicochemical characterization and, most importantly, in accurately predicting the biological activity (Pfister and Morbidelli, 2014). Furthermore, despite the administration of PEGylated G-CSF, a significant number of patients still develop neutropenia, suggesting a need for longer-lasting and more efficient versions of PEGylated G-CSF (Bartel et al., 1987; Lyman et al., 2010; Chan et al., 2011).

The second generation of site-specific PEGylation methods has been described for generating more homogenously PEGylated proteins (Pasut and Veronese, 2012; Dozier and Distefano, 2015). Over the last few years, maleimide chemistry has been utilized for the PEGylation of the thiol group of cysteine in therapeutic proteins. Given that this method has very high specificity for cysteine residues in the protein and there are only a few free cysteines in a protein, this technique has become the method of choice for PEGylation of therapeutics (Doherty et al., 2005; Rosendahl et al., 2005). This method also provides an opportunity to engineer more stable and improved variants of therapeutic proteins through addition, substitution, and deletion of cysteines. Earlier, we have published that the PEGylation of streptokinase (SK, a thrombolytic agent that dissolves fibrin blood clots) decreases antigenicity and increases *in vivo* half-life (Sawhney et al., 2016b). Subsequently, we showed that the PEGylation of truncated streptokinase enhanced plasmin resistance, prolonged half-life, improved fibrin clot-specificity, and reduced immune-reactivity compared to the native SK (Sawhney et al., 2016a). More recently, we have also shown that the site-specific PEGylation of microplasmin, a potential therapeutic for *in situ* stroke intervention, significantly enhanced functional half-life via decreasing antiplasmin-mediated inhibition. The positioned PEGylated microplasmin not only prolongs renal/metabolic clearance rates but also minimizes microplasmin-antiplasmin intermolecular interactions, consequently slowing the inhibitory reaction (Kaur et al., 2019).

The site-specific PEGylation has also been employed for the PEGylation of G-CSF. G-CSF contains one free cysteine (at position 17) and two disulfide bonds (Cys36-Cys42 and Cys64-Cys74). These disulfide bonds are critical for structural integrity and G-CSF activity; however, Cys17 could be mutated. Interestingly, Cys17 was also utilized for generating a PEGylated version of G-CSF (Veronese et al., 2007). Since the cysteine 17 is partially buried in a hydrophobic pocket, this method requires denaturation followed by PEGylation and subsequent protein renaturation. Thus, this cysteine is not suitable for site-specific PEGylation. It is worth mentioning that mutation of Cys17 to alanine or serine improves its stability (Ishikawa et al., 1992), permitting the insertion/substitution of cysteine at desirable positions to impart desired traits. Conventionally, 20 kDa PEG has been conjugated to G-CSF, but higher-molecular-weight PEG aldehydes were also conjugated. Interestingly, PEGylation with 30 kDa aldehyde PEG instead of 20 kDa (used in commercial PEGylated G-CSF) at N-terminal resulted in increased serum half-life and higher bioavailability (Zhai et al., 2009).

In the present study, we utilized a computational approach for identifying strategically defined solvent-accessible sites wherein cysteines could be introduced or substituted without affecting G-CSF activity. Solvent accessibility is essential for efficient and homogenous PEGylation. G-CSF PEGylation was carried out at these sites, using methoxy PEG maleimide moiety of different molecular weights. PEGylation and homogeneity of the reaction product were confirmed using various physicochemical methods. Finally, prolonged and enhanced activity was detected in the neutropenia mice model through neutrophils’ enumeration in mice blood.

## Materials and Methods

### Materials

All enzymes and reagents required for molecular biology were acquired from Fermentas (Waltham, MA, United States). Purification of plasmid and DNA from agarose gel was performed using kits available from Qiagen (Hilden, Germany). Expression host *Escherichia coli* strain BL21 (DE3) was procured from Novagen Inc. (Madison, WI, United States). The oligonucleotide primers used in the study for cloning and site-directed mutagenesis were custom synthesized from Integrated DNA Technologies (IDT), United States, and BioServe Biotechnologies (India) Pvt Ltd., G-CSF standards (09/136 and 12/188) were purchased from the National Institute for Biological Standards and Control (NIBSC). Commercially available G-CSF and its PEGylated versions were from Dr. Reddy’s Laboratories Ltd., Methoxy PEG maleimide reagents were purchased from JenKem Technology, United States. Methoxy PEG maleimide with catalog nos. A3115, A3116, and A3123 were used in this study. SP-Sepharose, Macro CAP SP, Capto SP ImpRes, Superdex-75 pg, and Superdex-200 pg resins and columns used for different chromatographic processes were procured from GE-Healthcare Life Science, Sweden. Zeba Spin Desalting Columns were purchased from Thermo Fisher Scientific, United States. Spectrophotometric analysis was performed on Perkin Elmer LAMBDA 35 UV/Vis spectrophotometer. All the reagents were of the highest available analytical grade. We used an AKTA Pure M (25M1) chromatographic system with Unicorn 7.3 software, version 7.3. Cyclophosphamide (C7397) was from Sigma-Aldrich. B.D. Vacutainer K3EDTA tubes were used to collect mice’s blood.

### Construction of G-CSF Variants and Their Expression

The human cDNA G-CSF sequence was retrieved from NCBI GenBank^1^ using accession number M13008.1. This sequence was codon-optimized for expression in *E. coli* using the OptimumGene^TM^ algorithm from GenScript, United States. The codon-optimized cDNA sequence of human G-CSF was cloned at the *Nde*I and *Hin*dIII sites of the expression vector pET23a using primers IMTSD1 5’ ATG ACG CCG CTG GGT CCG 3’ and IMTSD2 5’ CGG CTG TGC CAG GTG AC 3’. Free cysteine at position 17 of G-CSF was replaced with serine by site-directed mutagenesis to prevent unwanted PEGylation using primers SDM17a 5’ CTG CTG AAG TCT CTG GAA CAA 3’ and SDM17b 5’ TTG TTC CAG AGA CTT CAG CAG 3’. The cysteine substitution and or insertion variants were derived from the G-CSF variant in which cysteine 17 has been changed to serine. G-CSF variants were created using standard protocols of site-directed mutagenesis or by PCR using primers with desired changes for insertion or substitution of cysteine at a particular position in the coding sequence, as reported earlier (Kaur et al., 2019). For the Cys2 variant described in this manuscript, the following oligonucleotides were used: SDC2 5’ ATG TGT CCG CTG GGT CCG 3’ as forward and IMTSD2 5’ CGG CTG TGC CAG GTG AC 3’ as reverse primer. These cysteine variants were used to transform the BL21 (DE3) strain of *E. coli* for protein expression.

### Structure Modeling of G-CSF Variants

The structure assembly simulation of G-CSF was performed using the I-TASSER web server (Roy et al., 2010). This was performed to simulate lysine residues and cysteine substitution or insertion position on the G-CSF structure. The TM-align structural alignment program was also run to compare G-CSF Cys2 I-TASSER simulation to all the available structures in the PDB library.

### Determination of Solvent-Accessible Sites

We also utilized computational biology for identifying the regions wherein cysteines could be introduced or substituted for assisting maleimide-based PEGylation. Solvent accessibility was determined using webserver NetSurfP-2.0 (Petersen et al., 2009). This server provides the protein surface accessibility score and secondary structure predictions. The absolute surface accessibility values are a vital parameter to ascertain the solvent accessibility of the residues.

### Purification of G-CSF

The G-CSF and cysteine variants cloned in the T7 RNA polymerase inducible promoter-based expression vector pET23a were used to transform the BL21 (DE3) strain of *E. coli*. 0.5 mM isopropyl β-D-1-thiogalactopyranoside (IPTG) was used to induce protein in the form of inclusion bodies, which were then solubilized in 2 M urea. Washed inclusion bodies (IB) were solubilized in urea containing buffer (2 M urea in 50 mM Tris–HCl, pH 12.0) for 60 min. After centrifugation at 11,000 rpm for 20 min at 4°C, the supernatant containing the solubilized IBs was collected. The pH was adjusted to 8.0 by using glacial acetic acid. Partially unfolded protein was further subjected to a two-step refolding procedure using refolding buffers (50 mM Tris-Cl pH 8.0, 50 mM NaCl and 5 mM EDTA for 12–16 h followed by 25 mM sodium acetate pH 4.5, 50 mM NaCl, and 5 mM EDTA pH 8.0 for 6–8 h or till the pH of the sample reached 4.5) at 4°C with gentle stirring. Each refolding step is given 1–2 change of buffer for efficient refolding of the protein. Refolded protein was purified using cation exchange chromatography (CEC) on Capto SP ImpRes resins from GE-Healthcare Life Science, Sweden, equilibrated with 25 mM sodium acetate pH 4.5, 50 mM NaCl, and 5 mM EDTA. The G-CSF protein was eluted using 1 M Tris-Cl, pH 8.0.

### Conjugation of PEG to G-CSF and Purification of PEGylated G-CSF

In the case of the cysteine variants of G-CSF, the cation exchange purified protein was used to conjugate PEG of varying sizes. The protein was dialyzed in PEGylation buffer, i.e., sodium phosphate buffer (pH 7.5). The 5-fold molar excess of thiol-specific PEG was used to conjugate the Cys2 G-CSF variant in PEGylation buffer. The reaction was allowed to proceed at 20°C for 12–16 h with slow stirring. The PEGylation reaction was dialyzed against 25 mM sodium acetate pH 4.5 and 5 mM EDTA. The PEGylated species were purified by CEC using MacroCap SP resins or Capto SP ImpRes resins (from GE-Healthcare Life Science, Sweden), followed by size exclusion chromatography (SEC) using Superdex-75 pg or Superdex-200 pg column. The CEC purified PEGylated protein was loaded onto the Superdex column, preequilibrated with 25 mM sodium acetate pH 4.5, 50 mM NaCl and 5 mM EDTA and eluted in the same buffer. Fractions containing the purified protein were collected and desalted in formulation buffer [10 mM sodium acetate (pH 4.5), 5% sorbitol and 0.004% Tween 20, followed by filtration using 0.22 μm filter] and stored at −20°C.

### Analytical Characterization of PEGylated G-CSF Variants

All PEGylated and non-PEGylated derivatives were checked for their purity on 10.5% non-reducing SDS-PAGE and stained with Coomassie Brilliant Blue (CBB). Barium iodide staining was also used to visualizing the PEGylated G-CSF. To examine the PEGylated protein, barium iodide staining was performed, where PEG moiety is stained based on the formation of a barium iodide complex with PEG. The gel was treated with 5% barium chloride solution for 10 min and then with 0.1 M iodine solution for 5 min (Kurfurst, 1992; Zheng et al., 2007).

### Mass Spectrometry Analyses

The molecular mass of G-CSF, cysteine variants, and PEG conjugated variants were determined by Matrix-Assisted Laser Desorption Ionization Time of Flight Mass Spectrometry (MALDI-TOF) on an ABI SCIEX machine, model TripleTOF^®^ 5600/5600^+^.

### Circular Dichroism Spectroscopy

Circular dichroism (CD) spectroscopy was performed to investigate the secondary structure of G-CSF, cysteine variants, and PEG-conjugated variants. Far-UV CD spectra of wild-type and modified variants were recorded from 195 to 250 nm on the Jasco J-815 spectro-polarimeter at 25°C. Measurements of all the samples were performed at concentrations 0.2 mg/ml, using cuvettes of 0.1 cm path length.

### Formulation of G-CSF, Cysteine Variants, and PEG-Conjugated Cysteine Variants for Analyzing *in vivo* Bioactivity in a Neutropenia Mice Model

PEGylated cysteine variants were formulated in a formulation buffer consisting of 10 mM sodium acetate (pH 4.5), 5% Sorbitol, and 0.004% Tween 20, followed by filtration using a 0.22 μm filter. Protein samples were aliquoted for subsequent usage and stored at −20°C. Before administration in mice, samples were quantitated by NanoDrop (Thermo Scientific^TM^) and by running on 10.5% non-reducing SDS-PAGE gel. Quantitation was done using ImageJ software.

### *In vivo* Experiments on Neutropenia Mice

All the mice experiments were approved by the Institutional Animal Ethics Committee of Council of Scientific and Industrial Research-Institute of Microbial Technology (IAEC Approval no. IAEC/16/05 and IAEC/18/14). These experiments were performed according to the guidelines issued by the Committee for the Purpose of Supervision of Experiments on Animals (No.55/1999/CPCSEA) under the Prevention of Cruelty to Animals Act 1960 and amendments introduced in 1982 by the Ministry of Environment and Forest, Govt. of India. Mice were maintained and bred in the animal house facility of CSIR-Institute of Microbial Technology; 8–12-week-old male BALB/c mice were used in the current study for analyzing the biological activity of PEG-conjugated cysteine variants of G-CSF. Toward this, mice were acclimatized for a week, and neutropenia was induced in mice using an intraperitoneal injection of cyclophosphamide (200 mg/kg body weight of mice) as per standard procedures. Cyclophosphamide is an anti-cancer chemotherapy drug. This medication is classified as an alkylating agent and also used to induce neutropenia in laboratory animals (Scholz et al., 2009). To confirm neutropenia induction, blood was withdrawn and collected in K3EDTA Vacutainer tubes, and total leukocyte counts (TLC) were measured. TLC was determined using the HmX Hematology Analyzer from Beckman Coulter, and data were also confirmed by Leishman’s staining. One-day post-induction of neutropenia, sham (having formulation buffer without therapeutic protein), PEGylated G-CSF (commercially available), and the engineered cysteine PEGylated variants were independently administered as the single subcutaneous dose. To analyze the effect of increasing the molecular weight of PEG on neutrophil proliferation, 40 μg of each protein was administered as a single subcutaneous dose. The equimolar concentration of standard G-CSF and Cys2 40 kDa PEGylated G-CSF was administered to analyze the effect of the Cys2 variant on the day of cyclophosphamide treatment. To investigate the effect of the half-dose of Cys2 40 kDa PEGylated variant on neutrophil proliferation, mice were administered 1 mg/kg of mice weight of standard G-CSF or 0.5 mg/kg of mice weight of Cys2 40 kDa PEGylated G-CSF. After G-CSF treatment, the blood samples were withdrawn from the retro-orbital plexus, and TLC counts were determined every third day (3^*rd*^, 6^*th*^, 9^*th*^, 12^*th*^, and 15^*th*^ day).

### Statistical Analysis

GraphPad Prism 6 for Windows, Version 6.05, was used to plot animal experiment data. The plots represent scatter dot plot wherein data are means with SEM for three mice per group. Statistical significance was determined using two-way ANOVA multiple comparisons of the data. ^∗^indicates a *P*-value < 0.05, ^∗∗^indicates a *P*-value < 0.01, ^∗∗∗^indicates a *P*-value < 0.001, and ^****^indicates a *P*-value < 0.0001.

## Results

### Identification and Selection of Optimal Sites for Inserting and or Substituting Cysteine

With the overarching goal of creating PEGylated variants with prolonged biological activity, we utilized computational biology for identifying the regions wherein cysteines could be introduced or substituted for assisting maleimide based PEGylation. Toward this, we analyzed the structure of G-CSF for the identification of solvent-accessible sites, wherein PEGylation will not impede the activity and binding with G-CSF receptor. Available structure of G-CSF complexed with the ligand-binding region of the G-CSF receptor was used for this purpose (Aritomi et al., 1999; Tamada et al., 2006). It is important to note that all the cysteine substitution variants were derived from the G-CSF variant in which cysteine 17 has been changed to serine. This was done to ensure mono-PEGylation on single free thiol group of engineered cysteine in G-CSF. We reasoned that solvent accessibility would increase the efficiency of the PEG conjugation of G-CSF, besides shielding the protein from proteases and thereby increasing the *in vivo* half-life. Solvent accessibility was determined using webserver NetSurfP-2.0 (Petersen et al., 2009). This server provides the values for absolute surface accessibility. Detailed analysis with this server for G-CSF revealed that N- and C-terminal regions have unstructured solvent-accessible regions. Besides, we also observed that loop region between helix C and D is also unstructured and is solvent-accessible. Based on these considerations, several solvent-accessible sites that would not impede G-CSF’s biological activity were identified (listed in Table 1). We choose substitution of threonine with cysteine after the first amino acid methionine to provide a proof of concept. This variant (referred as Cys2 variant hereafter) was utilized for the rest of the studies presented in this manuscript. We also performed a structure assembly simulation of G-CSF using the I-TASSER web server (Roy et al., 2010; Figures 1A,B). Furthermore, we utilized the TM-align structural alignment program to compare G-CSF Cys2 I-TASSER simulation to all the structures in the PDB library, which confirmed the substitution of cysteine at position 2 does not affect the salient structural features of the G-CSF (Figure 1C).

**TABLE 1:** The preferred position for cysteine substitution or insertion for site-specific PEGylation of G-CSF^#^.

Proximal to Helix A Amino acid 1–10	T1, P2, L3, G4, P5, A6, S7, S8 MCT_1,_ T_1_CP_2,_ P_2_CL_3,_ L_3_CG_4,_ G_4_CP_5_, P_5_CA_6,_ A_6_CS_7_
Helix A Amino acid 11–39	E33, K34
AB loop Amino acid 40–70	K40, L61
Helix B Amino acid 71–91	Q90
BC loop Amino acid 92–99	P97, E98, L99, S_96_CP_97_, P_97_CE_98_, L_99_CG_100_
Helix C Amino acid 100–123	P101, E122, E123, P_101_CT_102_, M_121_CE_122,_ E_122_CE_123_
CD Loop Amino acid 124–142	P128, P138, L_124_CG_125_, M_126_CA_127_, Q_134_CG_135_, P_138_CA_139_
Helix D Amino acid 143–172	R146, R147, R169, H170, L171, A172, A_143_CF_144,_ R_146_CR_147,_ R_169_CH_170_, H_170_CL_171_, L_171_CA_172_, A_172_CQ_173_
Distal to Helix D Amino-acid 173–174	Q173, P174, Q_173_ P_174_C, Q_173_CP_174_

***^#^**Solvent accessibility was determined using webserver NetSurfP-protein surface accessibility and secondary structure prediction. This server provides the values for absolute surface accessibility, which was utilized to select solvent-accessible suitable sites for PEGylation.*

### Cloning, Expression, and Purification of the Cys2 Variant

**FIGURE 2:**
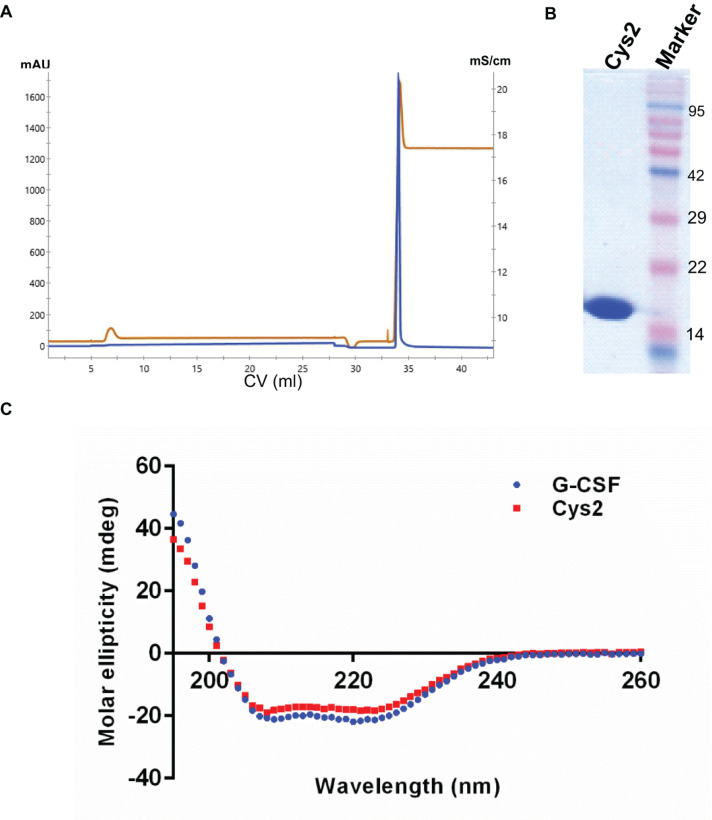
Purification and characterization of the G-CSF Cys2 variant. **(A)** Cation exchange chromatography profile of the Cys2 variant of G-CSF. Protein was eluted by 1 M Tris–HCl. Parameters such as absorbance at 280 nm and conductance have been represented with blue and brown lines, respectively. **(B)** The SDS-PAGE profile shows the general purity of the eluted Cys2 variant protein. **(C)** A comparison of far UV circular dichroic spectra of Cys2 cysteine variant and wild type G-CSF.

The codon-optimized cDNA sequence of human G-CSF was cloned in the expression vector pET23a. Free cysteine at position 17 was mutated to serine using site-directed mutagenesis to prevent unwanted PEGylation at Cys17. Threonine was substituted with cysteine at the N-terminal after the first methionine using standard methods. Both, the substitution of cysteine at position 2 (Cys2) and the mutagenesis of cysteine 17 to serine were confirmed using DNA sequencing. Cys2 variant was overexpressed in *E. coli*. Similar to the wild-type huG-CSF, this variant also resides in the inclusion bodies and was purified using denaturation with urea followed by two-step refolding and chromatographic purification (Figure 2A). SDS-PAGE analysis revealed that the purified Cys2 variant exhibits a single protein band at the right size (Figure 2B). Finally, CD spectroscopy was utilized to analyze the secondary structure of the Cys2 variant. This analysis suggested that cysteine substitution does not affect the overall secondary structure of the Cys2 variant (Figure 2C).

### Mono-PEGylation, Purification, and Characterization of Cys2 Variant

**FIGURE 3:**
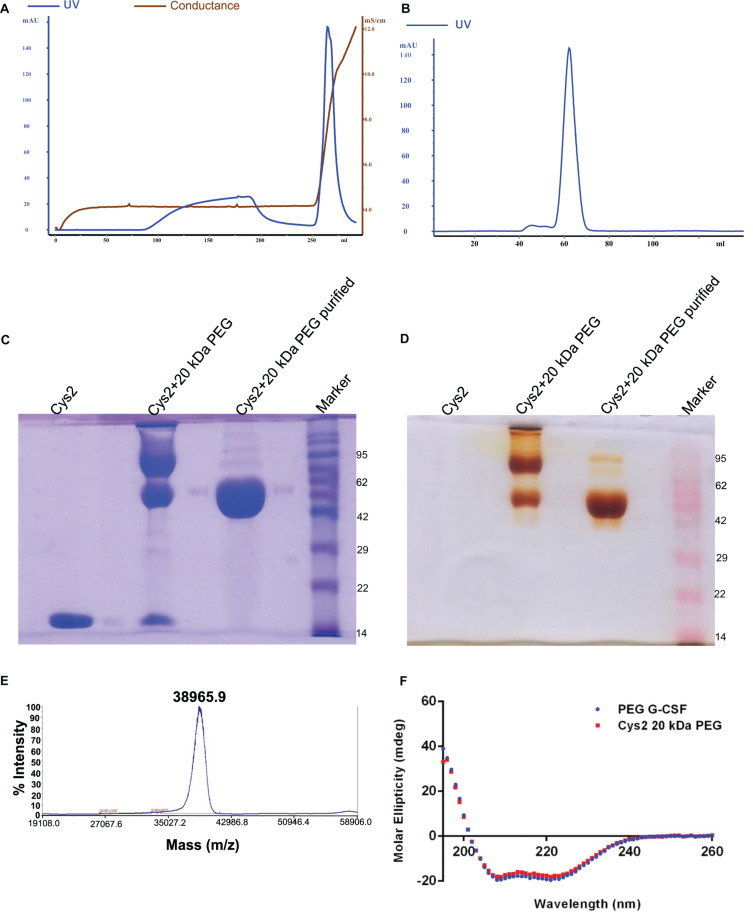
Purification and characterization of PEGylated Cys2 variant of G-CSF. **(A)** The first purification step (i.e., cation exchange chromatography, CEC) profile of the 20 kDa PEGylated Cys2 variant of G-CSF. **(B)** The second purification step (i.e., size exclusion chromatography, SEC) profile of CEC purified Cys2 variant protein in its PEGylated form. **(C)** Non-reducing SDS-PAGE profile stained with CBB showing the purity of purified PEGylated protein, wherein Lane 1 is the non-PEGylated cysteine variant, Lane 2 is the CEC-eluted peak fraction corresponding to PEG-conjugated Cys2 protein, Lane 3 is the SEC purified fraction, and Lane 4 is the Marker. **(D)** Same samples run on different gel and stained with barium iodide for staining specifically PEGylated protein. **(E)** The MALDI-TOF profile of purified Cys2 variant conjugated with 20 kDa PEG. **(F)** Comparison of far UV circular dichroic spectra of Cys2 variant conjugated with 20 kDa PEG and commercial available 20 kDa PEGylated G-CSF.

Purified Cys2 variant was conjugated with 20 kDa methoxy PEG maleimide using maleimide chemistry. The PEGylated species were purified by CEC using MacroCap SP resins or Capto SP ImpRes resins (from GE-Healthcare Life Science, Sweden), followed by SEC (Figures 3A-B). SDS-PAGE analysis followed by CBB staining confirmed PEGylation of the Cys2 variant (Figure 3C). These results also suggest high efficiency of PEGylation since the non-PEGylated species were present only in minute traces. We also utilized barium iodide staining to confirm the PEGylation. Barium iodide specifically stains PEG molecules with very high sensitivity and thus could resolve PEGylated species that are not visible with the CBB staining. Here again, we observed highly specific mono-PEGylation of the Cys2 variant with traces of multi-PEGylated species (Figure 3D). SEC revealed highly homogenous PEGylation of the Cys2 variant (Figures 3C-D). This is important since aldehyde based amino-terminal PEGylation produces a heterogeneous mixture of multispecies of PEGylated huG-CSF (Shekhawat et al., 2019). MALDI-TOF analysis also suggested the formation of highly homogenous mono-PEGylated Cys2 variant (Figure 3E). Next, we analyzed whether PEGylation of Cys2 variant has any effect on the secondary structure of the protein. Toward this, CD spectroscopy was utilized. We observed that the secondary structure of the Cys2 variant conjugated with 20 kDa methoxy PEG maleimide was similar to the commercially available PEGylated G-CSF drug (Figure 3F). In summary, these findings suggest that Cys2 variant of huG-CSF could be homogenously mono-PEGylated with high efficiency and exhibit similar physicochemical characterization to G-CSF drug.

### *In vivo* Biological Activity of PEGylated Cys2 Variant

**FIGURE 4:**
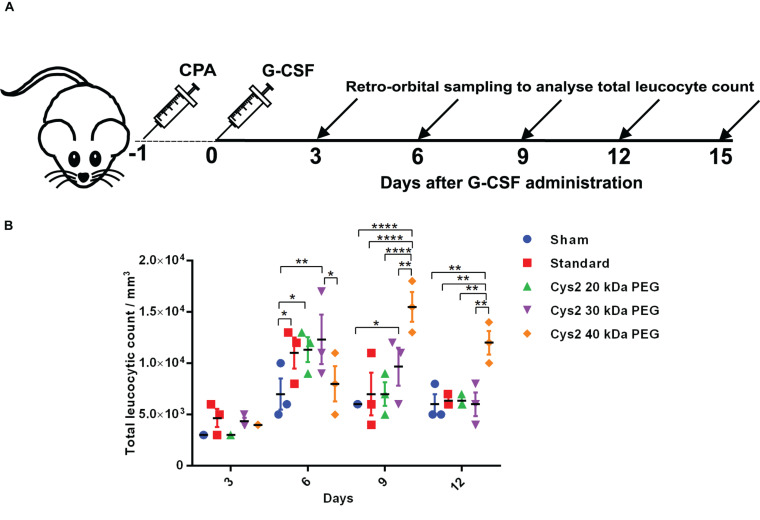
Biological activity of PEGylated variants in a neutropenia mice model. **(A)** Schematic depiction of the experimental procedure. CPA denotes cyclophosphamide. **(B)** Biological activity comparison of commercially available 20 kDa PEGylated G-CSF (standard) and Cys2 variant conjugated with 20 kDa, 30 kDa, and 40 kDa methoxy PEG maleimide in neutropenic mice. Total leucocytic counts (TLC) were determined following a single subcutaneous injection of 40 μg of G-CSF variants. The plots represent a scatter dot plot wherein data are means with SEM for three mice per group. Statistical significance was determined using two-way ANOVA multiple comparisons of the data. *indicates a *P*-value < 0.05, **indicates a *P*-value < 0.01 and ****indicates a *P*-value < 0.0001.

The above experiments suggested homogeneous mono-PEGylation of the Cys2 variant of huG-CSF. Next we analyzed whether the PEGylated variant is biologically active or not. Toward this, we established a mice model of neutropenia. Cyclophosphamide was administered intraperitoneally in 8–12-week-old mice for inducing neutropenia. Neutropenia was confirmed through the measurement of total leukocyte counts (TLC) on day 0. After the induction and confirmation of neutropenia, sham (having buffer only), commercially available PEGylated huG-CSF, and the mono-PEGylated Cys2 variant were independently administered as the single subcutaneous dose (summarized in the schematic provided in Figure 4A). After G-CSF treatment, the blood samples were withdrawn, and TLC counts were determined on 3^*rd*^, 6^*th*^, 9^*th*^, and 12^*th*^ day. We observed that the PEGylated Cys2 variant conjugated with 20 kDa PEG possesses similar biological activity to that of the commercially available PEGylated huG-CSF (Figure 4B). These observations suggest that Cys2 substitution in G-CSF and its PEGylation via maleimide chemistry do not hamper the activity of huG-CSF.

### Higher-Molecular-Weight PEGylation Leads to the Prolonged Activity of Cys2 Variant in Neutropenic Mice

**FIGURE 5:**
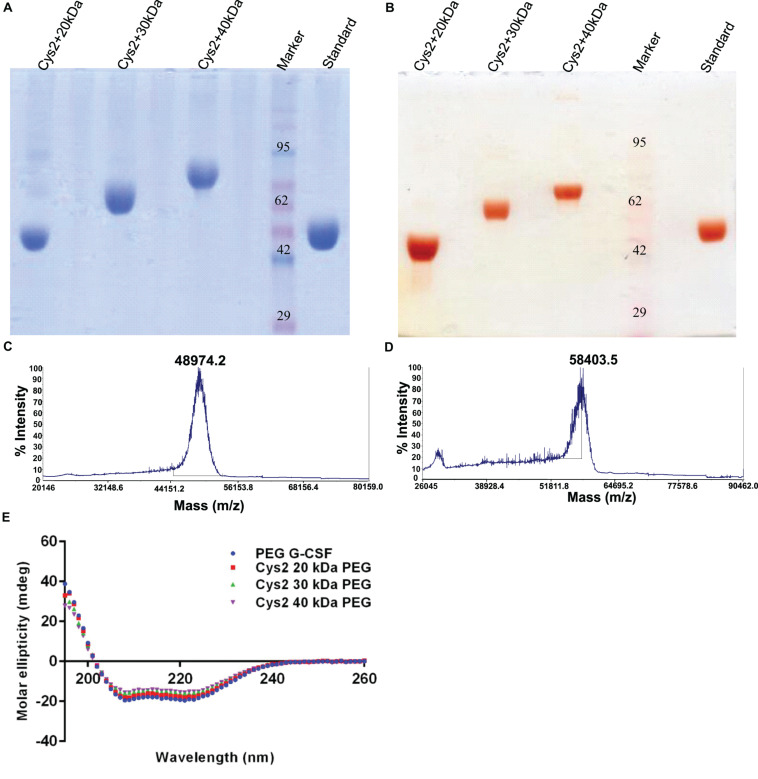
Characterization of PEGylated G-CSF Cys2 variant. **(A)** CBB-stained non-reducing SDS-PAGE analysis of purified higher-molecular-weight PEG-conjugated Cys2 variants. **(B)** Barium iodide-stained gel for purified protein samples to stain specifically PEGylated protein. **(C)** The MALDI-TOF profile of purified Cys2 variant conjugated to 30 kDa PEG and **(D)** 40 kDa PEG. **(E)** Comparison of far UV circular dichroic spectra of Cys2 variant conjugated with different sizes of PEG and commercially available 20 kDa PEGylated G-CSF.

After establishing that the PEGylation of the Cys2 variant does not hamper its activity, we analyzed whether conjugation of the Cys2 variant with higher-molecular-weight PEG improves and prolongs its biological activity. The Cys2 variant was conjugated with 30 kDa and 40 kDa methoxy PEG maleimide. The PEGylated Cys2 variant was purified as described earlier and analyzed on non-reducing SDS-PAGE using CBB staining (Figure 5A), barium iodide staining (Figure 5B), and MALDI-TOF mass spectrometry (Figures 5C-D). CD spectroscopy confirmed that PEGylation with 30 kDa and 40 kDa PEG does not alter the secondary structure of G-CSF (Figure 5E). Finally, we analyzed the biological activity of the Cys2 variant conjugated with 30 kDa and 40 kDa PEG. We observed that on sixth day post-administration, the TLC count was higher in the group that received the Cys2 variant conjugated with 30 kDa PEG. Importantly, we also observed that TLC counts in the groups receiving Cys2-30 kDa and Cys2-40 kDa conjugates were higher on the 9^*th*^ day as compared to sham, standard, or mice receiving the Cys2-20 kDa. Furthermore, the Cys2 variant conjugated with 40 kDa PEG even showed higher leukocyte recovery on the 12^*th*^ day, indicating prolonged biological activity (Figure 4B). These data clearly show that the G-CSF variant conjugated with higher-molecular-weight PEG possesses prolonged biological activity, perhaps due to longer *in vivo* retention of the Cys2-30 kDa and Cys2-40 kDa conjugates.

### Half-Dose of Cys2-40 kDa PEG Conjugate Has Improved and Longer Biological Activity Than the Full Dose of 20 kDa PEG Conjugate

**FIGURE 6:**
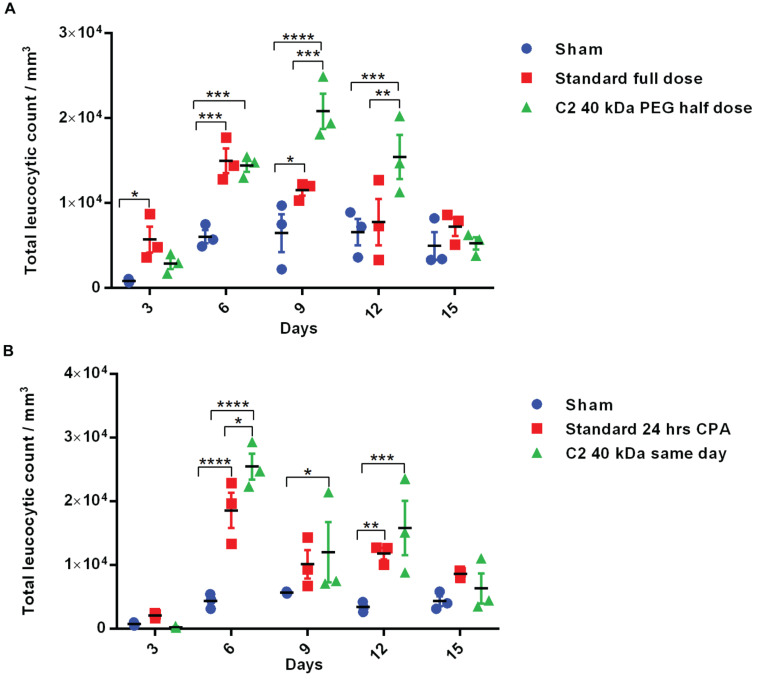
Biological activity of half-dose and same-day administration of PEGylated variants. **(A)** Biological activity profile of half-dose of Cys2 variant conjugated with 40 kDa methoxy PEG maleimide in neutropenic mice. **(B)** Biological activity profile of Cys2 variant conjugated with 40 kDa methoxy PEG maleimide administered same-day (after 8 h of CPA treatment) and compared with the standard administered, i.e., after 24 h of induction of neutropenia in mice. The plots represent a scatter dot plot wherein data are means with SEM for three mice per group. Statistical significance was determined using two-way ANOVA multiple comparisons of the data. *indicates a *P*-value < 0.05, **indicates a *P*-value < 0.01, ***indicates a *P*-value < 0.001 and ****indicates a *P*-value < 0.0001.

The above-described experiments suggest that the Cys2-40 kDa PEG conjugate possesses improved and longer biological activity than the Cys2-20 kDa PEG conjugate. Thus, we analyzed whether the half-dose (0.5 mg/kg mice body weight) of the Cys2-40 kDa PEG conjugate could compare for biological activity with the full dose (1 mg/kg mice body weight) of the standard 20 kDa PEG conjugate. Toward this, neutropenia was induced in mice as described above, and mice were treated with sham, a full dose of standard PEGylated G-CSF conjugated with 20 kDa PEG (1 mg/kg mice body weight) and half-dose (0.5 mg/kg mice body weight) of the Cys2-40 kDa PEG conjugate. Importantly, we observed that on day 6, the mice receiving a half-dose of the Cys2-40 kDa PEG conjugate and a full dose of 20 kDa PEGylated G-CSF have a comparable TLC. Importantly, on day 9 and 12, the proliferation of leukocytes was significantly higher in the group receiving the half-dose of the Cys2-40 kDa PEG conjugate compared to the group receiving the full dose of the standard PEGylated G-CSF conjugated with 20 kDa PEG (Figure 6A). These data suggest that even the half-dose of Cys2-40 kDa PEG conjugate has better and prolonged biological activity than the full dose of the standard PEGylated G-CSF.

### Same-day Administration of the Cys2-40 kDa PEG Conjugate Is Equally Effective as a Standard Regimen

One of the challenges in prophylactic administration of G-CSF during chemotherapy is that it must be administered 24 h after dispensing chemotherapeutic drugs to achieve adequate prophylactic protection from neutropenia. A same-day G-CSF substitute will be beneficial and as well as convenient for patients. As the administration of Cys2 variant conjugated with 40 kDa PEG has resulted in much longer biological activity, we checked whether the administration of the Cys2 40 kDa PEG conjugated variant at the day of neutropenia induction (mimicking the G-CSF treatment at the day of chemotherapy) could facilitate the recovery of leukocyte count in a neutropenic mice model. Interestingly, the same-day administration of the Cys2 variant conjugated with 40 kDa PEG has improved leucocytic recovery at day 6, 9, and 12 as compared to the standard PEGylated G-CSF conjugated with 20 kDa PEG administrated 24 h after cyclophosphamide treatment (Figure 6B).

## Discussion

huG-CSF is a beneficial and widely used therapeutic protein for several clinical conditions, including neutropenia, leukopenia, AIDS, sepsis, and also in patients undergoing bone marrow transplantation (Mehta et al., 2015). To increase its half-life, 20 kDa PEG aldehyde is conjugated to the first amino acid’s amino group. However, this method is associated with undesired non-specific PEGylation at the epsilon amino group of lysine residues besides the alpha-amino group (Veronese, 2001). Methoxy PEG maleimide has been utilized for site-specific PEGylation at the thiol group of cysteine residue. In the present study, we have employed a computational biology approach for the identification of solvent-accessible sites for the substitution or addition of cysteine residues in the protein for facilitating maleimide based site-specific PEGylation. Based on this analysis, we chose to substitute a cysteine residue at position 2 to generate the Cys2 variant. This variant was purified and conjugated with 20 kDa, 30 kDa, and 40 kDa PEG moieties. Using various physicochemical methods, we demonstrated that PEGylation at this solvent-accessible site does not measurably alter the structure of G-CSF. Furthermore, using the neutropenia mice model, we have shown that the 20 kDa methoxy PEG maleimide-conjugated Cys2 variant has similar leukocyte proliferative activity as that of the commercially available product. Importantly, we also demonstrate that conjugation of the Cys2 variant with 30 kDa and 40 kDa PEG prolongs its biological activity. Thus, through this site-specific, cysteine-dependent route, we can prepare engineered variants of G-CSF that are homogeneously PEGylated and exhibit clinically desirable traits that are a feature of next-generation G-CSF drug.

Accordingly, one of the significant findings of this study is the prolonged activity of the Cys2 variant of huG-CSF conjugated with 30 kDa and 40 kDa PEG compared to the 20 kDa PEG conjugated Cys2 variant. These are clinically relevant findings and agree with earlier studies wherein prolonged biological activity was correlated with the molecular weight of conjugated PEG moiety (Doherty et al., 2005; Sawhney et al., 2016b). Previous studies have shown that the addition of higher-molecular-weight N-terminal PEG to huG-CSF leads to decrease in its *in vitro* activity as monitored through the use of M-NFS-60 cell line-based assays and increase in *in vivo* circulation half-life (Zhai et al., 2009). It is important to note that in most of these studies, PEG molecules were in the range of 5–30 kDa, whereas in our study, we utilized PEG molecules from 20 kDa to 40 kDa. Similar results were observed in studies toward the design and development of PEGylated G-CSF (Arvedson et al., 2015). These results have led to the hypothesis that increasing PEG’s molecular weight hinders the binding of the huG-CSF with its receptor and, thus, is not ideal for *in vivo* use. Based on these considerations, commercial PEGylated huG-CSF is conjugated to 20 kDa PEG. Intriguingly, we observed that the conjugation of huG-CSF with 40 kDa results in prolonged *in vivo* activity. In fact, even the half-dose of the 40 kDa PEG molecule increased the proliferation of leukocytes till the 9^*th*^ day. In comparison, the commercially available 20 kDa PEG conjugated huG-CSF was virtually exhausted after the 6^*th*^ day of administration. In the light of the data presented in this study, the view that high molecular PEG hinders with *in vivo* binding of huG-CSF with its receptor needs to be revisited.

Another significant finding of the present study was that the same-day administration of the Cys2 variant conjugated with 40 kDa PEG effectively increased the leukocyte counts to desirable levels or more at the 6^*th*^, 9^*th*^, and 12th day. These findings are fascinating since such a method could pave the way for same-day administration of the prophylactic G-CSF variant. Currently, PEGylated G-CSF is administered 24 h after the administration of chemotherapeutic drugs. Thus, the patient needs to come back to the clinic for the administration of G-CSF (or its PEGylated form) 1 day after the administration of chemotherapy. We believe that innovations leading to same-day administration of chemotherapeutic and prophylactic agents could tremendously improve the outcome and be convenient for patients. We would also like to emphasize that we observed that in mice receiving same-day administration of huG-CSF-40 kDa PEG, the proliferation of leukocytes in neutropenic mice was considerably lower at the third day when compared to the administration of 20 kDa PEGylated G-CSF.

The production process for the currently available PEG conjugated huG-CSF relies upon amine-reactive PEGs (Molineux, 2004). Such PEGs react with the amine groups in a protein, including one at the N-terminal. Due to the underlying biochemistry, aldehyde PEG could also PEGylate the epsilon amino group of four lysine residues of huG-CSF. Some of these lysine residues are located in regions critical for binding with the G-CSF receptor (Yamasaki et al., 1994; Tamada et al., 2006). Additionally, non-specific PEGylation at lysine residues also hinders G-CSF interaction with its receptor leading to reduced activity. Thus, this approach is not ideal for G-CSF’s PEGylation, and other more specific PEGylation methods are required. In this direction, Cox and coworkers have utilized cysteine-reactive maleimide PEGs and developed methodology for site-specific PEGylation of G-CSF and other cytokines (Rosendahl et al., 2005). This method provides a unique opportunity to introduce cysteine at strategically defined sites. Building upon this methodology, we identified solvent-accessible PEGylation sites that could facilitate optimal PEGylation of G-CSF through thiol-reactive maleimide PEGs. We ensured that residues critical for G-CSF interaction with its receptor were deselected and suitable solvent-accessible sites that could be used for the PEGylation of G-CSF were identified. In all these constructs, cysteine 17 was mutated to serine to avoid non-specific PEGylation. These sites include an unstructured region at N-terminal, C-terminal, and the central loop regions. We utilized the N-terminal unstructured region and substituted cysteine at position 2. Next, we demonstrated that this site could be utilized for efficient and homogenous PEGylation. Several studies have suggested that the amine-reactive PEGs lead to heterogeneous PEGylation (Pasut and Veronese, 2012), whereas site-specific PEGylation in the current study resulted in highly homogenous PEGylation as demonstrated by non-reducing SDS-PAGE, barium iodide staining, and MALDI-TOF analysis. Several other sites were identified that could lead to specific and homogenous PEGylation of G-CSF, including the C-terminal unstructured region and the loop between helix C and D. It is important to emphasize that we also identified solvent-accessible sites in the CD loop region. This region is of particular interest since huG-CSF is O-glycosylation at threonine 133 in the CD loop (Kubota et al., 1990). This glycosylation protects the protein from degradation by proteases like neutrophil elastase (Carter et al., 2004). We believe that PEGylation at these solvent-accessible sites in the CD loop will increase biological half-life through imparting resistance to proteases and prevention from renal filtration simultaneously. However, studying the efficiency of these sites for PEGylation and afterward analyzing the effect on the biological activity is beyond the scope of this study. It would be carried out in the future as a separate study. In summary, here we have demonstrated that cysteine could be inserted/substituted at strategically defined solvent-accessible sites in G-CSF to facilitate site-specific PEGylation for the homogenous product with longer and improved *in vivo* biological activity.

## Conclusion

We have successfully designed G-CSF variants for site-specific PEGylation, which may provide a solution toward prolonged half-life and biological activity. The site-specific PEGylated variants were successfully expressed, purified, and characterized. Importantly, the administration of the engineered variant conjugated with 40 kDa PEG leads to improved and longer leukocyte proliferation in neutropenic mice, wherein neutropenia was induced by cyclophosphamide. Even at half the dose, the 40 kDa PEG conjugated G-CSF variant leads to enhanced and longer biological activity in the neutropenia mice model. Consequently, this variant could be administered at a lower dosage than the 20 kDa PEGylated G-CSF while retaining the same therapeutic efficacy. The site-specific higher-molecular-weight PEGylated variant also shows promise for the same-day administration of the G-CSF drug, which is a challenge in the current G-CSF therapy regimen in the treatment of chemotherapy-induced neutropenia. These engineered cysteine variants and their derivatives hold promise to become a next-generation G-CSF drug.

## Data Availability Statement

The raw data supporting the conclusions of this article will be made available by the authors, without undue reservation.

## Ethics Statement

The animal study was reviewed and approved by IAEC, CSIR-IMTECH.

## Author Contributions

SD conceived the study, helped in analysis of data, and wrote the manuscript. MK performed the experiments and analyzed the data. GS provided technical and administrative support to the project. He also helped in improving the manuscript. All authors contributed to the article and approved the submitted version.

## Conflict of Interest

The authors declare that the research was conducted in the absence of any commercial or financial relationships that could be construed as a potential conflict of interest.

## Footnotes

1 https://www.ncbi.nlm.nih.gov/genbank/
